# The Factors That May Predict Response to Rituximab Therapy in Recurrent Focal Segmental Glomerulosclerosis: A Systematic Review

**DOI:** 10.1155/2011/374213

**Published:** 2011-11-24

**Authors:** Carlos E. Araya, Vikas R. Dharnidharka

**Affiliations:** Division of Pediatric Nephrology, University of Florida College of Medicine, 1600 SW Archer Road, Room HD-214, Gainesville, FL 32610-0296, USA

## Abstract

Recurrence of FSGS occurs in 30–40% of allografts. Therapies for recurrence are not well established. We retrieved all published reports depicting kidney transplant recipients with focal segmental glomerulosclerosis (FSGS) recurrence, treated with rituximab, to determine factors associated with treatment response. We found 18 reports of 39 transplant recipients who received rituximab. By univariate analysis for two outcomes (no response versus any response), fewer rituximab infusions and normal serum albumin at recurrence were associated with treatment response. For 3 outcomes (no response, partial and complete remission), male gender, fewer rituximab infusions, shorter time to rituximab treatment, and normal serum albumin were associated with remission. Multivariate analysis for both models revealed that normal serum albumin at FSGS recurrence and lower age at transplant were associated with response. Rituximab for recurrence of FSGS may be beneficial for only some patients. A younger age at transplant and normal serum albumin level at recurrence diagnosis may predict response.

## 1. Introduction

Focal segmental glomerulosclerosis (FSGS) is a common cause of idiopathic nephrotic syndrome in childhood and comprises over one-third of such cases in adults. Patients with FSGS are at a substantial risk of progressing to end-stage renal disease (ESRD) requiring dialysis or renal transplant [1]. 

Following transplantation, 30 to 40% of patients with FSGS, due to a presumed underlying immune defect, will have recurrence of FSGS [2] which negatively impacts allograft survival. In contrast, FSGS secondary to mutations in genes encoding proteins expressed in the podocyte and elsewhere in the glomerular capillary wall has a low incidence of recurrence [3]. 

The underlying immune disorder leading to FSGS is not known, but is probably multifactorial. It has been hypothesized that FSGS is caused by a circulating glomerular permeability factor released by T cells [4]. Hence, plasmapheresis is the most commonly used therapy for established FSGS recurrence, but it is not always effective [5]. In the published literature, 70% of children and 63% of adults will have some response to plasmapheresis, especially if started early after diagnosis and repeated frequently [5]. 

Other investigators have proposed that B cells may be involved in the pathogenesis of FSGS through an abnormal cross-talk with T cells or by directly releasing the still unidentified permeability factor [6, 7]. Benz et al. treated with rituximab a patient with idiopathic thrombocytopenic purpura who also had steroid-dependent nephrotic syndrome [8]. After rituximab, the patient was able to discontinue oral corticosteroids without relapsing. Nozu et al. reported on a young boy with recurrence of FSGS in his allograft that developed posttransplant lymphoproliferative disorder (PTLD) [9]. After treatment that included rituximab, the PTLD resolved and the proteinuria abated. Other case reports and case series throughout the following years have documented both successes and failures in the treatment of recurrent FSGS with rituximab [9–26]. There are no prospective studies evaluating the effectiveness of rituximab in posttransplant FSGS. Yet, clinicians need better data for improved clinical decision making since rituximab use is not benign and may be associated with significant complications. The purpose of this study was to analyze all the existing reports and to determine if we can identify which factors are associated with remission of the clinical recurrence following rituximab treatment in posttransplant FSGS, thereby allowing for more focused therapy.

## 2. Materials and Methods

We retrieved reports of rituximab use in recurrent FSGS from PubMed using the search terms “nephrotic syndrome, treatment, and rituximab”. Our initial search yielded 96 articles. We then excluded review articles, reports of rituximab use in native kidney disease or in other disease recurrence after transplant. Only articles written in English were included. Our final study group included 18 reports describing 36 unique patients, plus 3 unpublished patients at our own center. One retrospective questionnaire study to members of the International Pediatric Nephrology Association that described 15 patients with recurrent FSGS was not included since some of the patients may already have been reported as case reports/series [27]. Data were extracted from each of these reports using a standardized questionnaire. The authors were individually contacted in order to obtain the following variables that were not always reported: recipient age at diagnosis and transplant, recipient gender, recipient race, donor age, donor gender, donor race, transplant source, time from transplant to recurrence, serum albumin level at recurrence, proteinuria at recurrence, number of plasmapheresis sessions, pretransplant plasmapheresis, immunosuppression used, presence of an acute rejection prior to or after the diagnosis of relapse, number of rituximab doses, time from transplant and relapse to rituximab administration, and the presence of PTLD. 

We defined clinical recurrence by the presence of nephrotic range proteinuria, since this was reported as present in all study reports and was the basis for treatment initiation. Not all cases had hypoalbuminemia or edema at the time of recurrence of FSGS. All but 5 patients underwent renal biopsies for diagnostic purposes. However, all patients had heavy proteinuria and were considered to have recurrence of FSGS. Complete response to therapy was defined as absence of proteinuria (protein/creatinine ratio of <0.3). Partial response to therapy was defined as more than 50% decline in proteinuria to a protein/creatinine level between 0.3 and 2. No response to therapy was defined when proteinuria failed to improve by at least 50% or remained above a protein/creatinine level above 2. 

Given the small number of reports in the study sample and uncertainty of distribution of these continuous variables in the population at large, we chose to analyze all continuous variables by the more rigorous Kruskal-Wallis nonparametric test. Continuous data are expressed as median and range. Categorical variables were analyzed by the Chi-square test of Fisher exact test, as appropriate, and are expressed as proportion (%). We handled response to therapy in two different ways: either as a two outcome variable (no response/any response) or three outcome variable (no response/partial response/complete response). The number of plasmapheresis sessions was entered into separate multivariate models either as a continuous variable or as a discrete ordered variable (<20 sessions, 20–50 sessions, >50 sessions).

Variables that showed a *P* value of <0.10 by univariate analysis were entered into stepwise backward logistic regression multivariate models to assess for association with clinical response. All statistical analyses were carried out using SAS 9.2 (Cary, NC, USA). A *P* value of <0.05 was considered as significant.

## 3. Results

The full cohort comprised 39 renal transplant patients from 18 published reports and our center's unpublished experience. The demographic characteristics for the patients are provided in Table 1. The individual contacting of corresponding authors helped us obtain complete data in all 39 subjects for most of the variables. The median age of the patients at the time of transplant was 18 years (range 5–48 years). Nineteen of the 39 patients were in the pediatric age group at the time of transplant. A negative genetic mutation for podocin was documented in 4 patients diagnosed with nephrotic syndrome at a young age and was not reported for the remaining patients. All but one patient were receiving maintenance immunosuppression with oral corticosteroids plus a combination of either tacrolimus/MMF or cyclosporine A/MMF. 

The clinical recurrence occurred early, with 61.5% of them presenting within the first week and 74.3% within the first month after transplant. Recurrence beyond one year of transplant occurred in only 10.2% of the cohort, in one case developing almost 10 years later. Posttransplant lymphoproliferative disorder (PTLD) occurred in 3 of the pediatric patients and in none of the adult patients. 

Plasmapheresis was used for the treatment of recurrent FSGS in 38 of the 39 patients with only one patient not receiving plasmapheresis. In 9 patients, plasmapheresis was initiated before transplant and continued after transplantation. 

All patients received rituximab ranging from 1 to 6 doses (median 4). The time from transplant to rituximab administration was median 210 days (range 4–3543 days). However, the “gap time” or the number of days from diagnosis of recurrence of FSGS to rituximab administration was shorter at median 149 days (range 3–1086 days). In most cases, rituximab was used as a late effort to achieve remission, which explains the prolonged gap time.

By univariate analysis for 2 outcomes (no response to therapy versus any response to therapy), a fewer number of rituximab infusions (*P* = 0.007) was associated with a higher frequency of response (Table 2). This suggests that patients who responded after the initial rituximab doses did not receive subsequent infusions. Also, a normal serum albumin level at the time of FSGS recurrence (*P* = 0.02) was associated with any response to rituximab therapy. The patients who improved with therapy were younger at the time of transplant (16 versus 30 years), but this was not statistically significant (*P* = 0.09). 

 The data were then analyzed for 3 outcomes (Table 3): no response, partial remission, and complete remission of the FSGS recurrence. Male gender was associated with a higher frequency of achieving remission (*P* = 0.01). A fewer number of rituximab infusions was again associated with response to therapy (*P* = 0.03). Furthermore, a shorter time to rituximab treatment following relapse (shorter “gap time”) was also significantly associated with response to therapy (*P* = 0.03). A normal serum albumin level at relapse was also associated with response to therapy (*P* = 0.05).

The age at diagnosis, recipient age at transplant, time to ESRD, time from transplant to relapse, transplant source, degree of proteinuria, number of plasmapheresis sessions, pretransplant plasmapheresis, immunosuppression used, and the presence of PTLD were not significantly associated with FSGS remission after rituximab administration. Age at transplant showed a borderline significance of *P* = 0.07 in a 3-outcome univariate model. 

We then fitted two stepwise backward logistic regression multivariate models, one each for the 2-outcome or 3-outcome models. We included those variables with complete data and a *P* value <0.10 in univariate analyses into the logistic regression models (number of rituximab doses, serum albumin at recurrence, age at transplant, male gender, and time from recurrence to rituximab). The models for the outcome of any response to therapy revealed that two variables were significantly associated with response after rituximab therapy. A normal serum albumin level at diagnosis of FSGS recurrence (point estimate 6.46, 95% CI 1.55–26.84, *P*  value = 0.01) and lower age at transplant (point estimate 0.92, 95% CI 0.85–0.98, *P*  value = 0.014) were the significant predictors. In the models for the 3 outcomes, the only two variables that again predicted a complete response or partial response over no response to therapy were a normal serum albumin level at FSGS recurrence (point estimate 4.82, 95% CI 1.54–15.02, *P*  value = 0.007) and a lower age at transplant (point estimate 0.89, 95% CI 0.82–0.96, *P*  value = 0.003). None of the other variables analyzed for the 2- or 3-outcomes models were found to be significant. 

Rituximab infusions were overall well tolerated. Only two investigators reported side effects attributed to rituximab administration in the posttransplant FSGS patients [20, 23]. One patient developed neutropenia thought to be due to rituximab and one other a severe anaphylactic reaction at the time of infusion. Another patient developed BK virus nephropathy 2 weeks after the third rituximab infusion and catheter-related severe sepsis two months after the last rituximab dose [15]. However, the patient had also received intravenous corticosteroids and a polyclonal antilymphocyte globulin for treatment of an acute rejection.

## 4. Discussion

The pathogenesis of primary FSGS is still unclear, and its recurrence after renal transplant remains a predicament. Proposed mechanisms include abnormalities in the immune system including T-cell dysfunction/dysregulation, abnormal cross-talk between T and B cells, cytokines, and an unidentified circulating glomerular permeability factor [28]. Recently, Wei et al. have identified serum soluble urokinase receptor (suPAR) as a circulating factor that may cause recurrent FSGS [29]. As a result, intensive therapies like plasmapheresis in combination with oral cyclophosphamide or high-dose cyclosporine are commonly prescribed but are not always successful and accelerated allograft failure ensues. 

Rituximab, a chimeric monoclonal antibody against CD20, was initially approved for the treatment of B-cell Non-Hodgkin lymphoma and is now used for the treatment of other hematological malignancies and autoimmune disorders. There have been multiple reports of patients with steroid-dependent and steroid-resistant nephrotic syndrome as well as posttransplant FSGS who have been treated with rituximab (for review see [30]). 

Overall, our data show that the response to rituximab is variable and less effective in posttransplant FSGS as compared to idiopathic nephrotic syndrome in the native kidney, where response rates are higher [30]. This finding could be explained by the fact that a large proportion of steroid-dependent and some of the steroid-resistant patients in the above studies had renal histology consistent with minimal change disease and not FSGS. The first prospective study evaluating rituximab in idiopathic nephrotic syndrome was performed by Guigonis et al. [31]. The study included 22 patients, 3 with histologic changes of FSGS. Two of the patients were treated with rituximab after remission had been achieved and one during relapse. The patient that was actively nephrotic failed to respond to therapy. Two other patients, actively nephrotic, did not benefit from rituximab. The investigators concluded that disease activity may impact the efficacy of rituximab. By definition, in posttransplant FSGS, all patients have active disease and heavy proteinuria which may explain why response to rituximab is lower than in primary FSGS. In addition, the study by Guigonis also noted that rituximab was more effective in patients on a combination of corticosteroids and a calcineurin inhibitor [31]. However, our analysis revealed that the type of immunosuppressive agent was not predictive of response to therapy. 

The short-term effects of rituximab in children with steroid-dependent nephrotic syndrome were evaluated by Ravani and colleagues in an open-label randomized controlled trial [32]. This study included mostly patients with renal histology consistent with minimal change disease, but 25% of patients in the intervention arm that received rituximab had a diagnosis of FSGS. At 3 months followup, children who received rituximab had less proteinuria, were receiving lower prednisone and calcineurin inhibitor doses, and had lower risk of relapse. However, patients with steroid-resistant or with steroid-dependent nephrotic syndrome on high-prednisone doses were excluded from this study. Furthermore, the outcomes were not analyzed based on renal histology making it even more difficult to extrapolate the data to the posttransplant FSGS patient population. 

In our present analysis, we did find that patients with a normal serum albumin at the time of recurrent FSGS diagnosis were more likely to achieve remission. The normal serum albumin level was documented prior to initiating plasmapheresis with albumin replacement. There are several possible reasons for this association. A normal serum albumin level may imply an earlier stage of disease, when rituximab may have a better chance of working. Alternatively, it may imply a milder disease phenotype. It is important to mention that patients with FSGS secondary to an underlying immune defect and patients with FSGS due to mutations in podocyte proteins may have different response to therapy. Furthermore, there have been an increasing number of podocyte-expressed genes associated with steroid-resistant nephrotic syndrome in both children and adults. In the current cohort, *NPHS2* mutation analysis was performed and found to be negative in only 4 patients. Analyses for other slit-diaphragm-associated proteins were not carried out in these or any other of the reported patients. Hence, it is possible that some of the patients may have carried an unidentified podocyte protein mutation. 

In the initial reports by Nozu and Pescovitz, rituximab induced complete remission of nephrotic syndrome in a patient with PTLD and another who developed Epstein-Barr-virus-driven diffuse large cell lymphoma. Follow-up investigators reported treatment failure with rituximab in patients without PTLD. In the existing reports, there are only 3 patients treated for malignancy with rituximab. Complete remission of proteinuria was induced in two and partial remission in one of the patients. We found no association between the presence of PTLD and response to rituximab therapy. 

So, how could rituximab be effective in some cases of recurrent FSGS? It could be assumed that CD20+ cells play some role in the pathogenesis of posttransplant FSGS. For instance, Dantal et al. treated four patients with relapse of focal glomerulosclerosis after transplantation using an (non-protein A) anti-Ig affinity column and a protein A column [7]. The two procedures were effective in depleting the relapsing patients' plasma of the factor capable of altering the albumin permselectivity of isolated glomeruli in vitro. The investigators concluded that immunoglobulins have a role in the recurrence of nephrotic syndrome and suggested that the responsible factor may be bound to an immunoglobulin. Furthermore, CD20 is expressed in a fraction of T cells and this population could be targeted with rituximab as well with a resulting alteration in T-cell and B-cell cross-talk [33]. Rituximab can also inhibit nuclear factor kappa B (NF-*κ*B) pathway in CD20+ cells [34]. NF-*κ*B regulates cytokine expression which was reported by Sahali et al. to be altered in patients with nephrotic syndrome. A recent study suggests that rituximab may act through a B-lymphocyte-independent mechanism by directly regulating podocyte activation [35]. Investigators found that rituximab binds to sphingomyelin phosphodiesterase acid-like 3b (SMPDL-3b) in podocyte lipid rafts and prevents its downregulation when exposed to sera from patients with recurrent FSGS. Rituximab also preserved acid sphingomyelinase enzymatic activity at normal levels, essential for the organization of receptors and signaling molecules in the podocyte. Furthermore, rituximab appears to prevent podocyte actin remodeling through stabilization of SMPDL-3b. 

In summary, multiple immunological mechanisms or even a direct podocyte effect could be involved in the response following rituximab administration in some patients. Overall, complete remission of the proteinuria was achieved in 17/39 (43.5%) of patients reported to date and this may be an overestimation. The efficacy of rituximab in recurrence of FSGS cannot be estimated based on the current published reports due to possible publication bias, as patients without response may not have been reported in the literature and due to the lack of controlled prospective trials. Also, remission of proteinuria was induced in some patients several months after rituximab administration, while they were still receiving other interventions. 

In summary, adjuvant therapy with rituximab for recurrence of FSGS may be beneficial in only some patients and the response will likely be evident after the initial medication doses. A younger age at transplant and a normal serum albumin level at the time of recurrence seem to predict response to treatment. Clearly, prospective controlled trials are needed to prove efficacy and safety of rituximab in posttransplant FSGS.

## Figures and Tables

**Table 1:** Demographic characteristics of renal transplant patients with recurrence FSGS that received rituximab. Values expressed as median (range) or proportion (%).

Variable	Results
Age at diagnosis (years)	6 (1–40)
Age at transplant (years)	18 (5–48)
Time to end stage renal disease (years)	3 (0.16–19)
Recipient male	22/39 (56.4)
Deceased donor transplant	26/39 (66.7)
Time to relapse of FSGS (days)	3 (1–3513)
Serum albumin at relapse (g/dL)	2.5 (1.2–4.4)
Proteinuria at relapse (g/day)	6.2 (1.1–97.4)
Diagnosis of PTLD	3/39 (7.7)
Number of plasmapheresis treatments	21 (0–133)
Number of rituximab doses	4 (1–6)
Complete response to therapy	17/39 (43.5)
Time to response from rituximab (months)	2 (0.63–12)

**Table 2:** Univariate analysis for the 2-outcome model of any response versus no response to rituximab therapy. Values expressed as median (range) or proportion (%).

Variable	Any response (*n* = 25)	No response (*n* = 14)	*P* value
Age at diagnosis (years)	5 (1–33)	12 (1.9–40)	0.07
Age at transplant (years)	16 (5–48)	30 (5.5–48)	0.09
Male gender	16/25 (64)	6/14 (42.8)	0.31
Time to end stage renal disease (years)	3 (0.16–19)	4 (0.5–12)	0.42
Time to relapse (days)	2 (1–3513)	22 (1–828)	0.13
Deceased donor source	16/25 (64)	10/14 (71.4)	0.73
Serum albumin at relapse	3.25 (1.4–4.4)	2.3 (1.2–3.3)	0.02
Proteinuria at relapse (g/day)	8 (1.1–97.4)	7.64 (2.4–20)	0.98
Plasmapheresis	21 (0–100)	27 (9–133)	0.36
Pretransplant plasmapheresis	7/25 (28)	2/14 (14.4)	0.45
Posttransplant plasmapheresis	24/25 (96)	14/14 (100)	1.00
Rituximab doses	3 (1–6)	4 (2–6)	0.007
Time from transplant to rituximab (days)	150 (4–3543)	276 (65–1050)	0.29
Time from relapse to rituximab (days)	136 (3–1086)	179 (57–1048)	0.32
Diagnosis of PTLD	3/25 (12)	0/14 (0)	0.54
Cyclophosphamide use	2/25 (8)	2/14 (14.4)	0.85
Tacrolimus use	21/25 (84)	9/14 (64.3)	0.84
Mycophenolate mofetil use	24/25 (96)	13/14 (92.8)	1.00

**Table 3:** Univariate analysis for the 3-outcome model of complete, partial response, and no response, to rituximab therapy. Values expressed as median (range) or proportion (%).

Variable	Complete response (*n* = 17)	Partial response (*n* = 8)	No response (*n* = 14)	*P* value
Age at diagnosis (years)	5 (1–33)	7.5 (2–30)	12 (1.9–40)	0.12
Age at transplant (years)	13 (5–48)	22.5 (8–41)	30 (5.5–48)	0.07
Male gender	14/17 (82.4)	2/8 (25)	6/14 (42.8)	0.01
Time to end stage renal disease (years)	3 (0.16–19)	3 (0.6–9)	4 (0.5–12)	0.65
Time to relapse (days)	1 (1–3513)	8 (1–150)	22 (1–828)	0.12
Deceased donor source	11/17 (64.7)	6/8 (75)	10/14 (71.4)	1.00
Serum albumin at relapse (g/dL)	2.7 (1.4–4.4)	3.2 (2–4)	2.3 (1.2–3.3)	0.05
Proteinuria (g/day)	10.25 (1.1–97.4)	5.8 (2–14)	7.64 (2.4–20)	0.19
Plasmapheresis				0.43
<20 treatments	7/17 (41.2)	2/8 (25)	6/14 (42.8)	
21–50 treatments	7/17 (41.2)	3/8 (37.5)	2/14 (14.4)	
>50 treatments	3/17 (17.6)	3/8 (37.5)	6/14 (42.8)	
Pretransplant plasmapheresis	6/17 (35.3)	1/8 (12.5)	2/14 (14.4)	0.37
Posttransplant plasmapheresis	16/17 (94.1)	8/8 (100)	14/14 (100)	1.00
Rituximab doses	2 (2–6)	3 (1–5)	4 (2–6)	0.03
Time from transplant to rituximab (days)	120 (4–3543)	343 (10–1095)	276 (65–1050)	0.08
Time from relapse to rituximab (days)	62 (3–927)	271 (9–1086)	179 (57–1048)	0.03
Diagnosis of PTLD	2/17 (11.8)	1/8 (12.5)	0/14 (0)	0.41
Cyclophosphamide use	1/17 (5.9)	1/8 (12.5)	2/14 (14.4)	0.81
Tacrolimus use	13/17 (76.5)	8/8 (100)	9/14 (64.3)	0.18
Mycophenolate mofetil use	16/17 (94.1)	8/8 (100)	13/14 (92.8)	1.00
